# Environment-Friendly Zinc Oxide Nanorods-Grown Cellulose Nanofiber Nanocomposite and Its Electromechanical and UV Sensing Behaviors

**DOI:** 10.3390/nano11061419

**Published:** 2021-05-27

**Authors:** Lindong Zhai, Hyun-Chan Kim, Ruth M. Muthoka, Muhammad Latif, Hussein Alrobei, Rizwan A. Malik, Jaehwan Kim

**Affiliations:** 1 CRC for Nanocellulose Future Composites, Inha University, Incheon 22212, Korea; duicaofei@naver.com (L.Z.); kim_hyunchan@naver.com (H.-C.K.); mwongelinruth@gmail.com (R.M.M.); mlatif8482@gmail.com (M.L.); 2 Department of Mechanical Engineering, Prince Sattam bin Abdul Aziz University, AlKharj 11942, Saudi Arabia; h.alrobei@psau.edu.sa; 3 Department of Metallurgy and Materials Engineering, University of Engineering and Technology, Taxila 47050, Pakistan; rizwanmalik48@yahoo.com

**Keywords:** cellulose nanofiber, zinc oxide, nanocomposite, electromechanical property, UV sensing

## Abstract

This paper reports a genuine environment-friendly hybrid nanocomposite made by growing zinc oxide (ZnO) nanorods on cellulose nanofiber (CNF) film. The nanocomposite preparation, characterizations, electromechanical property, and ultraviolet (UV) sensing performance are explained. CNF was extracted from the pulp by combining the 2,2,6,6-tetramethylpiperidine-1-oxyl radical (TEMPO) oxidation and the aqueous counter collision (ACC) methods. The CNF film was fabricated using doctor blade casting, and ZnO nanorods were grown on the CNF film by seeding and by a hydrothermal method. Morphologies, optical transparency, mechanical and electromechanical properties, and UV sensing properties were examined. The nanocomposite’s optical transparency was more than 80%, and the piezoelectric charge constant d_31_ was 200 times larger than the CNF film. The UV sensing performance of the prepared ZnO-CNF nanocomposites was tested in terms of ZnO concentration, UV irradiance intensity, exposure side, and electrode materials. A large aspect ratio of ZnO nanorods and a work function gap between ZnO nanorods and the electrode material are essential for improving the UV sensing performance. However, these conditions should be compromised with transparency. The use of CNF for ZnO-cellulose hybrid nanocomposite is beneficial not only for electromechanical and UV sensing properties but also for high mechanical properties, renewability, biocompatibility, flexibility, non-toxicity, and transparency.

## 1. Introduction

Organic and inorganic functional nanocomposites combine advantages of the individual materials that surpass parental material properties. They can achieve high mechanical strength, electrical conductivity, thermal conductivity, antibacterial, gas barrier, flame retardancy, electromagnetic shielding, optical transparency, energy harvesting, and actuating properties [1]. Cellulose nanofiber (CNF) is an outstanding organic material composed of nano-sized cellulose fibrils with a high aspect ratio [2,3,4,5,6,7]. The width of CNF is typically in the range of 5–20 nm, and its length is typically up to several micrometers. Cellulose molecules can form microfibrils during the biosynthesis process by forming inter- and intra-molecular hydrogen bonds [4]. CNF extracted from plants by the top-down approach has excellent properties: not only renewability, biodegradability, abundance, low price, and light weight but also high optical transparency, outstanding mechanical properties, and low thermal expansion coefficient [6]. Thus, CNF can be a building block of future materials applied for structural composites, coatings, cosmetics, 3D printing, sensors, soft actuators, flexible electronics, energy devices, and flexible displays [8,9,10,11,12,13].

Inorganic nanomaterials can improve the functional properties of organic-inorganic functional nanocomposites. The inorganic nanomaterials can be either blended or coated with/on the polymer nanocomposites [14,15]. Owing to its benefits in terms of wide bandgap (3.37 eV), high exciton binding energy (60 meV), ultraviolet (UV) response, optical transparency, highly electrical conductivity, and piezoelectricity, zinc oxide (ZnO) has been widely studied [16,17]. ZnO is broadly used in electronic devices, hybrid diodes, energy harvesters, piezoelectric devices, field-effect transistors, gas sensors, photovoltaics, and UV sensors [18,19,20,21,22,23,24,25]. It can be quickly grown on various substrates, including metal, glass, silicon, sapphire, plastics, polymers, and cellulose [20,26,27,28,29,30,31,32].

With increasing interest in wearable devices, the ZnO-cellulose hybrid composite is attractive because it can bridge ZnO’s functional properties and renewability and flexibility of cellulose. Recently ZnO nanorods grown on paper and cellulose have been reported [33,34,35,36,37,38]. A flexible and transparent cellulose-ZnO hybrid nanocomposite was prepared by direct ZnO seeding and hydrothermal ZnO nanorod growth on a regenerated cellulose film [35,36]. A zinc oxide nanolayer was uniformly formed on a regenerated cellulose film using a solution-based hydrothermal process, which shows a drastic improvement of its electromechanical behavior [37]. However, they used regenerated cellulose, which was prepared using special solvents, such as LiCl/DMAc [39]. It is essential to use CNF film instead of regenerated cellulose in cellulose-ZnO hybrid nanocomposites because the particular solvent usage can be eliminated.

Thus, this research aimed to prepare a genuine environment-friendly ZnO-CNF nanocomposite (ZCN) by growing ZnO nanorods on a CNF film according to the hydrothermal method. By using CNF instead of regenerated cellulose, the genuine environment-friendly ZnO-cellulose nanocomposite was prepared. Since CNF possesses numerous hydroxyl groups on its surface, ZnO can be efficiently anchored on the CNF film surface. This paper illustrates the CNF isolation, CNF film preparation, and the ZnO nanorods growth on the CNF film. The ZnO nanorods were grown by adopting the seeding process, followed by the hygrothermal process [37]. The morphologies, optical transparency, mechanical, electromechanical, and UV sensing properties of the prepared ZCN were investigated.

## 2. Materials and Methods

### 2.1. Materials

Hardwood (HW) pulp was received from Hansol Paper and Pulp Co. (Jeonju, Korea). HW bleached kraft pulp in dried pad form is a combination of Aspen and Poplar, and its alpha-cellulose (α) content is 85.7%, and viscosity is 14.6 cPs. 2,2,6,6-tetramethylpiperidine-1-oxyl radical (TEMPO, 98%), sodium bromide (NaBr, 99%), sodium hypochlorite (NaClO, 15%), and hydrochloric acid (HCl, 37%) were purchased from Sigma-Aldrich St. Louis, MO, USA, and Sodium hydroxide (NaOH, 98%) was purchased from Daejung Chemical, Busan, Korea. They were used to oxidize HW pulp further to extract CNF. Zinc acetate dihydrate (Zn(CH_3_COO)_2_·2H_2_O, reagent grade 98%) was purchased from Sigma-Aldrich, and ethyl alcohol anhydrous (C_2_H_5_OH, purity 99.5%) was purchased from Daejung Chemical. Zinc nitrate hexahydrate (Zn(NO_3_)_2_·6H_2_O, reagent grade 98%) and hexamethylenetetramine (HMT, (CH_2_)_6_N_4_, reagent grade 99%) were purchased from Sigma-Aldrich. All other chemicals used were analytical-reagent-grade (Purity > 99%) and used as received.

### 2.2. CNF Extraction and CNF Film Fabrication

The CNF was extracted by the TEMPO oxidation and aqueous counter collision system (ACC, ACCNAC-100, CNNT, Suwon, Korea) combined method, which has been reported previously [40]. The TEMPO oxidation acts as a pretreatment process for the ACC process. In brief, the HW pulp was cut into small pieces and swelled in deionized (DI) water for one day before disintegrating by a food mixer for 10 min. After that, TEMPO, NaBr, and DI water were added to the swollen HW pulp suspension. To start the oxidization process, the NaClO was added into the mixture and stirred at room temperature. A pH meter (Orion Star A211, Thermo Scientific, Waltham, MA, USA) was used to monitor the mixture’s pH value, maintaining it at 12 by adding NaOH. To stop the TEMPO oxidation reaction, 0.5 M HCl was added into the mixture and adjusted the pH value to 7. Finally, the oxidized cellulose pulp was washed with DI water to remove the chemical residues using a 90 μm mesh sieve. The TEMPO-oxidized pulp was further pulverized using the ACC system. The pulp was passed through a pair of diamond nozzles in a collision chamber with 200 MPa, such that a pair of aqueous solution jets collide against each other, resulting in an aqueous CNF suspension. The number of ejections passing through the nozzles was called “pass” As the number of passes increases, the CNF size decreases. TEMPO oxidation 60 min and ACC 30-pass-treated CNF was an optimum condition from the previous research [40], and the same condition was used in this research. The CNF suspension was degassed using a centrifuge machine (Supra 22K, Hanil Scientific Inc., Incheon, Korea) with 5000 rpm, 1 h.

**Figure 1:**
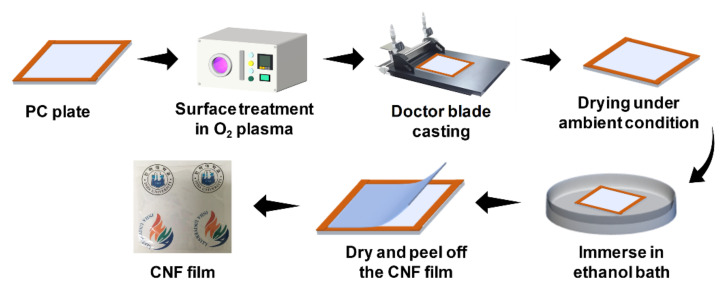
Schematic of CNF film fabrication process.

The CNF film was prepared using a doctor blade casting, as shown in Figure 1. A polycarbonate (PC) substrate was used for the CNF film casting. The PC substrate, where its boundaries were covered with the polyimide tape, was treated with an oxygen plasma using an oxygen-plasma treatment system (FEMTO Science, CUTE, Hwaseong-si, Korea) for 20 s to slightly increase its hydrophilicity of the PC substrate. The CNF suspension was cast on the plasma-treated PC plate using a doctor blade and dried in a cleanroom. After drying, the pristine CNF film was immersed in an ethanol bath for separating the pristine CNF film from the PC substrate. After evaporating ethanol, the pristine CNF film was peeled off from the PC substrate.

### 2.3. ZnO-CNF Nanocomposite Fabrication

**Figure 2:**
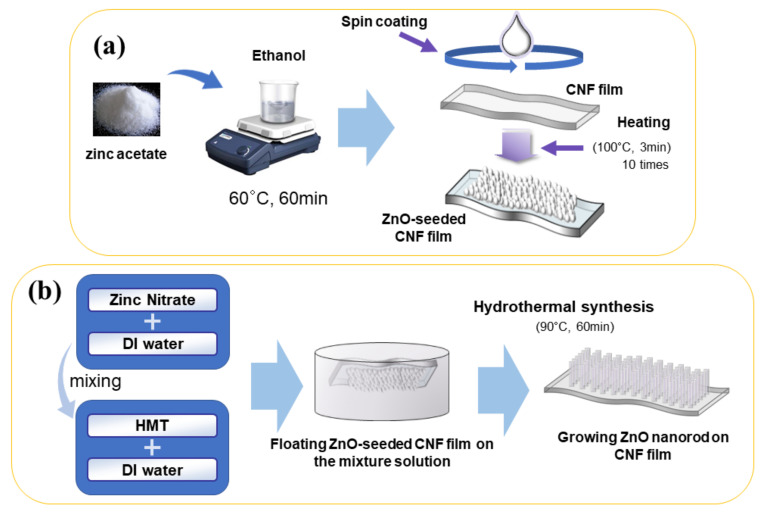
Schematic of ZnO-CNF nanocomposite fabrication process: (**a**) seeding process and (**b**) nanorod growing process.

The previously reported two-step process was adopted for growing ZnO nanorods on the CNF film: ZnO nanoparticles seeding and the ZnO nanorod growing [35]. In brief, the CNF film was fixed on a silicon wafer to maintain its flatness. Zinc acetate was dissolved in ethanol by stirring under 60 °C for 1 h, and the solution temperature was maintained at 60 °C to prevent ZnO crystal extraction. The solution was then spin-coated on the CNF film, followed by drying at 100 °C. This process was repeated 10 times with 3 min duration to form a dense ZnO seeding layer on top of the CNF film. After that, the ZnO-seeded CNF film was annealed at 100 °C for 30 min. Figure 2a shows the ZnO seeding process.

For growing ZnO nanorods, zinc nitrate aqueous solution and HMT aqueous solution were mixed. After the ZnO-seeded CNF film was floated on the surface of zinc nitrate aqueous solution, the solution was heated up to 90 °C for 1 h. As the nanorods grew, the CNF film color was changed from transparent to hazy then perfectly white. After growing ZnO nanorods, the nanocomposite was dried at room temperature. By controlling the chemical concentration, 25 mM and 50 mM of ZnO growing solutions were used to fabricate two ZCNs: 25 mM ZCN and 50mM ZCN. Figure 2b shows the schematic of the ZCN fabrication process.

### 2.4. Characterizations

A UV-2501PC UV-Vis spectrometer (Shimadzu, Kyoto, Japan) was used to analyze the transparency of the pristine CNF film and ZCNs. Field-emission scanning electron microscopy (FESEM) (S-4000, Hitachi, Matsuda, Japan) was used to investigate the morphologies of the pristine CNF film and ZCNs.

The mechanical and electromechanical properties were measured using a tensile test system, which consists of a load cell (UU-K010, Dacell, Nami-Myeon, Korea), a servo motor controller, a picoammeter (Keithley 6485, Tektronix, Beaverton, OR, USA), an environmental chamber, and LabVIEW software (National Instruments, Austin, TX, USA)) with a personal computer [41]. Aluminum electrodes were deposited on both sides of specimens by a thermal evaporation system (SHE-6D-350T, Samhan, Paju, Korea). Thin copper wires were connected to the electrodes and grounded to release space charges accumulated on the electrodes during the deposition. The electromechanical property was found by measuring the piezoelectric charge constant, d_31_, in the tensile test system. The specimens were subjected to a tensile load in its length direction, and the induced current was measured across the thickness direction from the electrodes using the picoammeter. The induced current can be converted into a charge per unit electrode area, and d_31_ can be obtained as:

(1) $$d_{31}={\left(\frac{\partial D_{3}}{\partial T_{1}}\right)}_{E}=\frac{\mathrm{Induced}\;\mathrm{charge}\;\mathrm{per}\;\mathrm{unit}\;\mathrm{electrod}\;\mathrm{area}}{\mathrm{Applied}\;\mathrm{in}-\mathrm{plane}\;\mathrm{normal}\;\mathrm{stress}}\;[C/N]$$

### 2.5. UV Sensing Test

**Figure 3:**
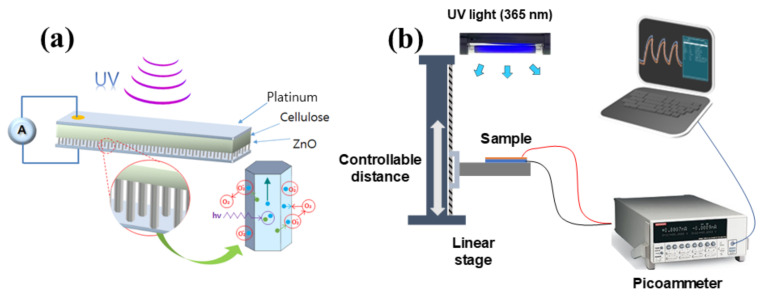
(**a**) Schematic of UV sensing ZCN and (**b**) UV-sensing test setup.

Figure 3a shows the schematic of UV sensing from ZCN. The ZCN was cut into 1.7 × 1.7 cm^2^, and then platinum or indium tin oxide (ITO) was coated with 1.5 × 1.5 cm^2^ size using a sputtering system (K575X, Quorum Technologies Ltd., Lewes, UK) to form electrodes on both side of the specimens. Thin copper wires were attached to both electrodes using a conductive silver paste and then coated with a thin laminate film to protect the ZnO layer. Before proceeding with the UV sensing test, the specimen was attached on a linear stage such that the distance between the UV light source and the specimen can be adjusted (see Figure 3b). A UV light lamp (PL-S 9W/2P BLB, Philips, Eindhoven, The Netherlands) of 365 nm wavelength was used as the light source. The UV irradiance intensity was measured using a UV meter (UV-A Meter, Kuhnast, Wächtersbach, Germany), which has the highest sensitivity at 360 nm wavelength. UV irradiance intensities of 1, 1.5, 3, and 5 mW/cm^2^ were applied to the specimens. For evaluating the UV sensing performance, the induced current was measured using the picoammeter. The UV irradiance was exposed to the cellulose side and ZnO side, and the exposure side effect was also investigated.

## 3. Results

### 3.1. Optical Transparencies and Morphologies

**Figure 4:**
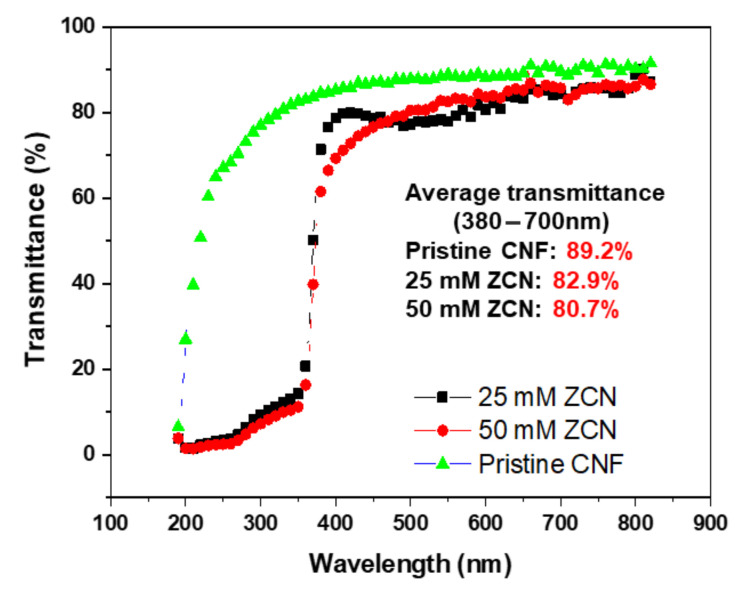
Optical transparencies of the pristine CNF film and ZnO-CNF nanocomposites.

Figure 4 shows the transparency of the prepared ZCNs and the pristine CNF film. The pristine CNF film showed the highest transparency of 89.2% in the visible light range, and the ZCNs exhibited high transparencies of 82.9% and 80.7% for the 25 mM and 50 mM ZnO concentrations. Within the UV wavelength range up to 370 nm, the ZCNs mostly block UV light, indicating that the ZnO nanorods adsorb most UV lights.

**Figure 5:**
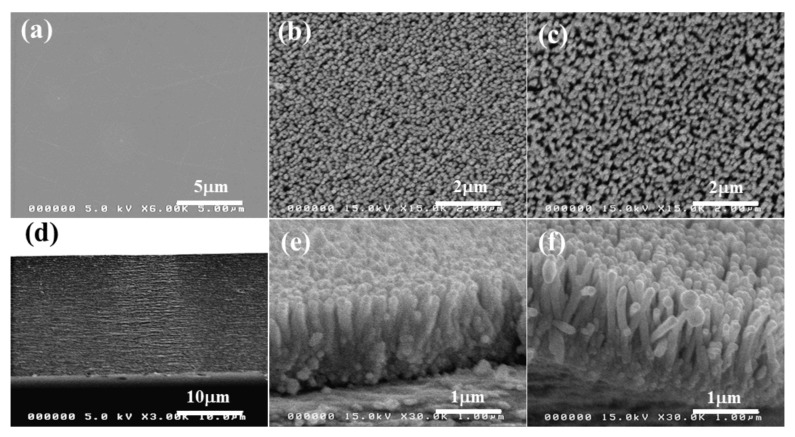
Surface (**a**–**c**) and cross-sectional (**d**–**f**) FESEM images: (**a**,**d**) the pristine CNF film, (**b**,**e**) 25 mM ZCN, (**c**,**f**) 50 mM ZCN.

Figure 5 shows the surface and cross-sectional morphologies of the pristine CNF film and ZCNs. The pristine CNF film surface is very smooth, and its cross-section exhibits a layered structure similar to the regenerated cellulose [42]. The ZnO nanorods were successfully grown vertically from the surface of CNF film, and their lengths of the 25 mM and 50 mM specimens are 500 nm and 800 nm. The vertically grown ZnO nanorods are beneficial for improving optical transparency and electromechanical properties. The ZnO nanorod morphologies are similar to the previous report [35]. The ZnO nanorod diameter was similar to the previous result, 110 nm [35]. Lower optical transparency of the 50 mM ZCN was attributed to the longer ZnO nanorods than the 25 mM ZCN.

### 3.2. Mechanical and Electromechanical Properties

**Figure 6:**
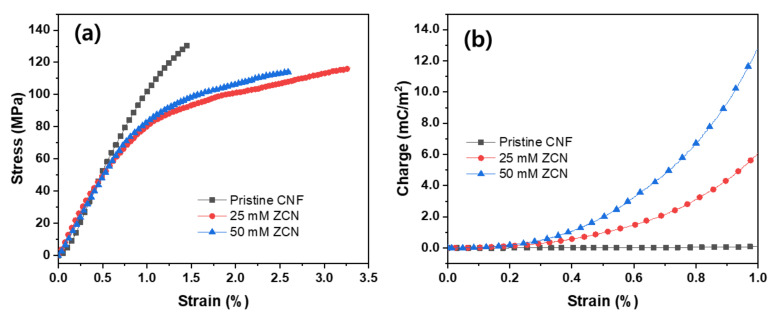
(**a**) Stress-strain curves and (**b**) induced charge curves of the pristine CNF film and ZCNs.

The mechanical and electromechanical properties of the pristine CNF and ZCNs were measured. Figure 6a shows the stress-strain curves of the specimens. The yielding occurred around 0.8–1.2% strain, and by fitting the slopes of the stress-strain curves within the elastic region, Young’s moduli were found. The results are listed in Table 1. The tensile strength (St) of the pristine CNF film was 131.4 MPa, which is a bit larger than the regenerated cellulose film (120 MPa, no-stretching) [42]. The Young’s modulus of the pristine CNF film was 12.8 GPa, much larger than the regenerated cellulose film (5.3 GPa, no-stretching). The CNF pristine film’s mechanical properties increase might be associated with the CNF alignment in the pristine CNF film. Thus, it is beneficial to use the pristine CNF film instead of the regenerated cellulose film for the ZCN.

After ZnO nanorod growing, the tensile strength of ZCNs ranged 115–116 MPa, and Young’s modulus was in the range of 9.6–9.7 GPa, a bit less than the pristine CNF film. The decreased mechanical properties might be due to the material mismatch between the CNF film and the grown ZnO nanorods. Note that the thickness of the ZnO nanorod layers was 0.5–0.8 μm, and the CNF film thickness was 20 μm. The grown ZnO nanorods cannot resist under the tensile load because they were grown perpendicular to the CNF film. Thus, although the ZnO layer thickness was smaller than the CNF film, the overall mechanical properties of ZCN were decreased.

Figure 6b shows the charge-strain curves of the pristine CNF film and ZCNs. Strain-induced charges increased with increasing the ZnO concentration within the elastic region. The piezoelectric charge constant, d_31_, of the pristine CNF film was 0.22 pC/N. This value is lower than the regenerated-cellulose electro-active paper (EAPap) without stretching, 3.4 pC/N [42]. Note that the d_31_ of the regenerated cellulose EAPap drastically increased up to 16–17 pC/N as the stretching ratio increased to 2.0 [42,43]. The d_31_ values of the 25 mM and 50 mM ZCNs were 26 and 48.8 pC/N. The maximum d_31_ of the 50 mM ZCN is 14 times larger than the regenerated cellulose EAPap, and 200 times larger than the pristine CNF film. The remarkable electromechanical property is attributed to the dipolar orientation of ZnO nanorods and surface piezoelectricity between the CNF film and the ZnO nanorods.

### 3.3. UV Sensing Test

**Figure 7:**
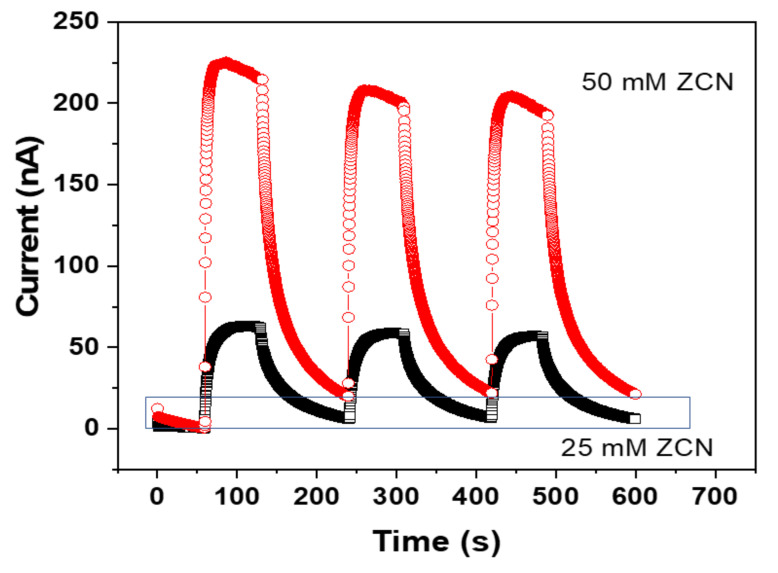
Induced current outputs of ZCNs with different ZnO concentrations.

The prepared ZCNs were UV-sensing-tested. The ZnO concentration effect on the UV sensing was investigated. Figure 7 shows the induced current from the ZCNs depending on the ZnO concentration when the UV light was on and off under 5 mW/cm^2^ UV irradiance. As the UV light was on, the current output from ZCNs sharply increased and saturated within 20–30 s. The 50 mM ZCN, however, gradually decreased after the saturation. After the UV light was off, the current output of ZCNs immediately decreased and returned to the nearly initial level after 120 s. Note that the current outputs are repeatable. The 50 mM ZCN exhibited almost four times higher current output than the 25 mM ZCN, which might be associated with the ZnO nanorods’ length. Since bigger ZnO nanorods in the 50 mM ZCN (800 nm) than the 25 mM ZCN (500 nm) give a larger surface area, more oxygen molecules can be attracted on the ZnO nanorods, such that larger current output can be obtained from the 50 mM ZCN than the 25 mM ZCN.

**Figure 8:**
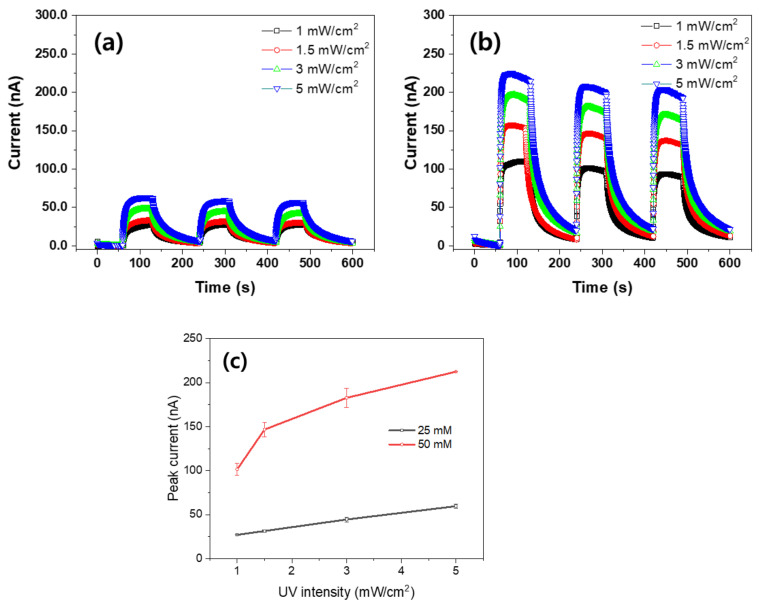
Induced current outputs of ZCNs: (**a**) 25 mM ZCN, (**b**) 50 mM ZCN, and (**c**) the UV irradiance intensity effect.

Figure 8a,b show the induced current outputs of ZCNs under the UV irradiance change. By controlling the distance between the UV light source and the specimens, the UV irradiance intensity was measured using the UV-A meter, and the intensity levels were 1, 1.5, 3, and 5 mW/cm^2^. Both 25 mM and 50 mM ZCNs were tested under the same condition. As the UV irradiance turned on and off with increasing the UV irradiance intensity, the current outputs followed the intensity signals and the maximum current outputs increased. Figure 8c shows the maximum current outputs of ZCNs with the UV irradiance change. The 25 mM ZCN exhibited a linear response, whereas the 50 mM ZCN represented a nonlinear response, which might be due to its large current output.

**Figure 9:**
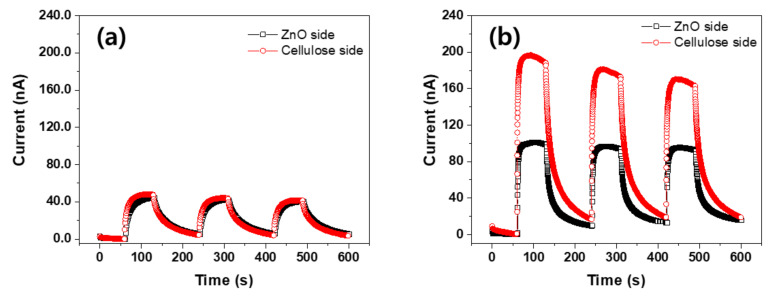
Current outputs of ZCNs with different UV irradiate sides: (**a**) 25 mM ZCN and (**b**) 50 mM ZCN.

Since the ZCN has two different sides, the UV exposure side effect was investigated by irradiating UV light onto the ZnO nanorods side and cellulose side. Figure 9 represents the induced current outputs of ZCNs by exposing UV irradiance to ZnO nanorods side and cellulose side for the 25 mM ZCN (a) and 50 mM ZCN (b). The test was performed under 3 mW/cm^2^ UV irradiance intensity. In the 25 mM ZCN, the current output from the cellulose side was slightly larger than the ZnO nanorods side. However, the 50 mM ZCN was different: the current output from the cellulose side was almost twice higher than the ZnO nanorods side. The CNF film is transparent, and the interface boundary between the CNF film and the ZnO seed layer is smoother than the ZnO nanorods side. The cellulose side scattered the UV irradiance less than the ZnO nanorods side, such that more current output occurred from the cellulose-side exposure [35].

**Figure 10:**
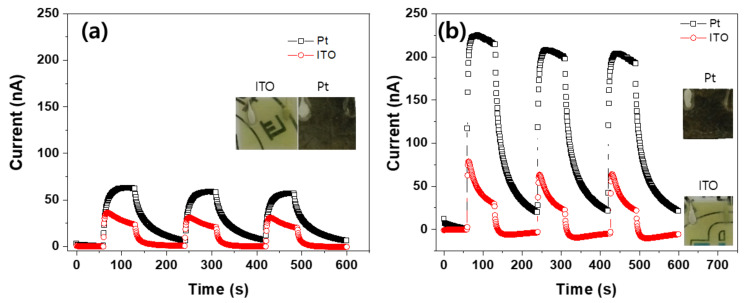
Platinum and ITO electrodes’ effect on the ZCN: (**a**) 25 mM ZCN and (**b**) 50 mM ZCN.

So far, Pt was coated on both sides of ZCNs to form electrodes. To investigate the electrode material effect on the UV sensing performance, a transparent electrode material, ITO, was coated on both sides of the ZCNs and compared with the Pt electrode. Figure 10 shows the current output of the 25 mM ZCN (a) and the 50 mM ZCN (b) for both electrode materials. Inset photographs show the images of the Pt- and ITO-coated ZCNs. Note that ITO is known to be transparent at 365 nm in contrast to Pt. Thus the ITO-coated ZCN is beneficial for achieving a transparent UV sensor. The Pt electrode ones showed larger current output than the ITO electrode cases in 25 mM and 50 mM ZCNs. The Pt-electrode-induced current was 1.8 times larger than the ITO electrode, and the 50 mM ZCN was 3 times larger. This phenomenon might be associated with the work function difference between the electrode and ZnO nanorods: ZnO work function is 4.3 eV, Pt is 5.7 eV, and ITO is around 4.3 eV [44]. The large work function gap between the ZnO and the electrode induced high current output under the same UV irradiance intensity. Since the work function of ITO electrode is almost the same as the ZnO, the ITO electrode did not show large current outputs. The Pt electrode on the ZnO nanorods could correspond to Schottky contact, which results in a large forward current and very low reverse bias. Additionally, the conductivity of the ITO electrode is lower than the Pt electrode. Note that the ITO electrode’s current outputs dropped quickly after turning on the UV light. It might be because the oxygen atoms incorporated into the ITO electrode decrease the oxygen vacancies and then give rise to the ITO electrode’s high resistivity, which resulted in fast current decay.

**Figure 11:**
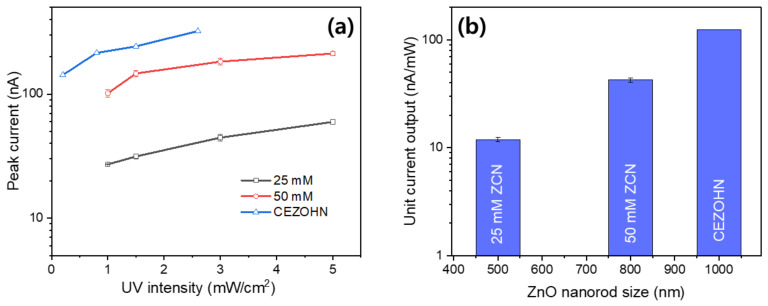
Current output comparison of ZCNs and CEZOHN: (**a**) peak current outputs with UV intensity changing and (**b**) the maximum unit current output with the ZnO nanorods’ length.

The UV sensing performance of ZCN was compared with the previously reported cellulose-ZnO hybrid nanocomposite (CEZOHN) [35]. CEZOHN is a ZnO-nanorod hybrid nanocomposite grown on a regenerated cellulose film. Figure 11a shows the peak current outputs of the ZCNs compared with the CEZOHN as the UV irradiation intensity changes. The current outputs were normalized with the electrode areas, and the ZCNs were recapped from Figure 8c. The peak current outputs increased with the UV irradiation intensity, and the CEZOHN exhibited higher UV sensing performance than the ZCNs. The high performance of UV sensing was investigated with the ZnO nanorod length. The ZCNs used the same ZnO seeding and growing process as the CEZOHN. Note that the CEZOHN used a 50 mM concentration of zinc nitrate aqueous solution for growing the ZnO nanorods. Figure 11b represents the unit current output of ZCNs and CEZOHN with the length of the nanorods. The unit current output was calculated from the maximum peak current outputs divided by the UV irradiation intensity, which turned out to be nA/mW. The ZnO nanorods’ length of CEZOHN was 1 μm, and the ZC was 500 nm and 800 nm. Table 2 represents the photosensitivities of the prepared ZCNs comparing with CEZOHN. The peak current increased from 59.69 ± 2.57 to 212.53 ± 9.09 nA for the 25 mM and 50 mM ZCNs, and their unit current output increased from 11.94 ± 0.51 to 42.51 ± 1.82 nA/mW/cm^2^. It is clearly shown that the current output is strongly related to the ZnO nanorods’ length. Thus, a large aspect ratio of ZnO nanorods is crucial for improving the UV sensing performance. A significant work function difference between ZnO nanorods and the electrode material should be made for performance improvement. However, the large aspect ratio of ZnO nanorods and considerable work function electrode material could deteriorate the ZCN’s transparency. Thus, they should be compromised with the transparency of ZCN. Nevertheless, the use of CNF for ZCN is beneficial for high mechanical properties, renewability, biocompatibility, flexibility, non-toxicity, and transparency.

## 4. Conclusions

In this study, the environment-friendly ZnO-CNF nanocomposites were fabricated by growing ZnO nanorods on the CNF film using two-step methods: seeding and nanorod growing by the hydrothermal method. The CNF extraction, the film casting, the ZnO nanorod growing, and all characterization were performed. The tensile strength and Young’s modulus of the CNF film (120 MPa, 12.8 GPa) were higher than the regenerated cellulose film (120 MPa, 5.3 GPa). Thus, it is beneficial to use the CNF film instead of the regenerated cellulose film to form the nanocomposites. Transparency of the ZnO-CNF nanocomposites slightly decreased from the pristine CNF film but still maintained more than 80%. The SEM images exhibited that the ZnO nanorods were grown uniformly on the CNF film surface, and the length of the nanorods was 500 nm and 800 nm for 25 mM and 50 mM ZnO concentration nanocomposites. After growing ZnO nanorods on the CNF film, the electromechanical property was increased more than 200 times compared to the pristine CNF film. As the ZnO concentration increased, the electromechanical property increased.

The UV sensing performance of the prepared ZnO-CNF nanocomposites was tested in terms of ZnO concentration, UV irradiance intensity, exposure side, and electrode materials. When increasing the ZnO concentration and UV irradiance intensity, the induced current output also increased. When the cellulose side was exposed to the UV irradiance, the induced current was higher than the ZnO nanorods-side exposure. Due to the transparency and smooth surface of the CNF film, it scattered UV irradiance less than the ZnO nanorods side. Regarding the electrode materials, the Pt electrode generated larger current output than the ITO electrode, which is associated with the work function difference between the electrode material and the ZnO nanorods.

The UV sensing performance of the ZnO-CNF nanocomposite and the previously reported regenerated cellulose-based ZnO hybrid nanocomposite was compared. The current output from the regenerated cellulose-based one was higher than the ZnO-CNF nanocomposites, attributed to the ZnO nanorods’ length. When increasing the length of the nanorods, the current output also increased. The high aspect ratio of ZnO nanorods in conjunction with the work function gap between ZnO nanorods and the electrode material are essential to improve the UV sensing performance, although they should be compromised with the transparency. The use of CNF for ZnO-cellulose hybrid nanocomposites is beneficial not only for electromechanical and UV sensing properties but also for high mechanical properties, renewability, biocompatibility, flexibility, non-toxicity, and transparency.

## Figures and Tables

**Table 1:** Mechanical and electromechanical properties of the pristine CNF and ZCNs.

Sample	E (GPa)	St (MPa)	d_31_ (pC/N)
Pristine CNF film	12.8 ± 0.1	131.4 ± 3.7	0.22 ± 0.1
25 mM ZCN	9.6 ± 1.4	116.0 ± 12	26 ± 5.6
50 mM ZCN	9.7 ± 0.3	115.0 ± 13.7	48.8 ± 11.7
Regenerated cellulose EAPap [36]	5.3	120	3.4

**Table 2:** Photosensitivities of the ZCNs compared with CEZOHN.

Sample	ZCNs		CEZOHN
	25 mM ZCN	50 mM ZCN	
Peak current (nA/cm^2^)	59.69 ± 2.57	212.53 ± 9.09	142.5
Unit current output (nA/mW/cm^2^)	11.94 ± 0.51	42.51 ± 1.82	124.0
ZnO nanorod size (nm)	500	800	1000
Response time (s)	70	35	25

## Data Availability

The data presented in this study are available on request from the corresponding author.
